# Dysregulation of FGFR signalling by a selective inhibitor reduces germ cell survival in human fetal gonads of both sexes and alters the somatic niche in fetal testes

**DOI:** 10.1093/humrep/dez191

**Published:** 2019-11-17

**Authors:** K Harpelunde Poulsen, J E Nielsen, H Frederiksen, C Melau, K Juul Hare, L Langhoff Thuesen, S Perlman, L Lundvall, R T Mitchell, A Juul, E Rajpert-De Meyts, A Jørgensen

**Affiliations:** 1 Department of Growth and Reproduction, Copenhagen University Hospital (Rigshospitalet), Blegdamsvej 9, 2100 Copenhagen, Denmark; 2 International Research and Research Training Centre in Endocrine Disruption of Male Reproduction and Child Health (EDMaRC), Blegdamsvej 9, 2100 Copenhagen, Denmark; 3 Department of Obstetrics and Gynaecology, Hvidovre University Hospital, Kettegård Alle 30, 2650 Hvidovre, Denmark; 4 Department of Gynaecology, Copenhagen University Hospital (Rigshospitalet), Blegdamsvej 9, Copenhagen 2100, Denmark; 5 MRC Centre for Reproductive Health, The Queen’s Medical Research Institute, University of Edinburgh, 47 Little France Crescent, Edinburgh EH16 4TJ, UK

**Keywords:** human fetal testis / human fetal ovary / *ex vivo* culture / FGF9 signalling / gonocytes / oogonia / gonadal sex differentiation / initiation of meiosis / somatic niche formation

## Abstract

**STUDY QUESTION:**

Does experimental manipulation of fibroblast growth factor 9 (FGF9)-signalling in human fetal gonads alter sex-specific gonadal differentiation?

**SUMMARY ANSWER:**

Inhibition of FGFR signalling following SU5402 treatment impaired germ cell survival in both sexes and severely altered the developing somatic niche in testes, while stimulation of FGF9 signalling promoted Sertoli cell proliferation in testes and inhibited meiotic entry of germ cells in ovaries.

**WHAT IS KNOWN ALREADY:**

Sex-specific differentiation of bipotential gonads involves a complex signalling cascade that includes a combination of factors promoting either testicular or ovarian differentiation and inhibition of the opposing pathway. In mice, FGF9/FGFR2 signalling has been shown to promote testicular differentiation and antagonize the female developmental pathway through inhibition of WNT4.

**STUDY DESIGN, SIZE, DURATION:**

FGF signalling was manipulated in human fetal gonads in an established *ex vivo* culture model by treatments with recombinant FGF9 (25 ng/ml) and the tyrosine kinase inhibitor SU5402 (10 μM) that was used to inhibit FGFR signalling. Human fetal testis and ovary tissues were cultured for 14 days and effects on gonadal development and expression of cell lineage markers were determined.

**PARTICIPANTS/MATERIALS, SETTING, METHODS:**

Gonadal tissues from 44 male and 33 female embryos/fetuses from first trimester were used for *ex vivo* culture experiments. Tissues were analyzed by evaluation of histology and immunohistochemical analysis of markers for germ cells, somatic cells, proliferation and apoptosis. Culture media were collected throughout the experimental period and production of steroid hormone metabolites was analyzed in media from fetal testis cultures by liquid chromatography–tandem mass spectrometry (LC-MS/MS).

**MAIN RESULTS AND THE ROLE OF CHANCE:**

Treatment with SU5402 resulted in near complete loss of gonocytes (224 vs. 14 OCT4^+^ cells per mm^2^, *P* < 0.05) and oogonia (1456 vs. 28 OCT4^+^ cells per mm^2^, *P* < 0.001) in human fetal testes and ovaries, respectively. This was a result of both increased apoptosis and reduced proliferation in the germ cells. Addition of exogenous FGF9 to the culture media resulted in a reduced number of germ cells entering meiosis in fetal ovaries (102 vs. 60 γH2AX^+^ germ cells per mm^2^, *P* < 0.05), while in fetal testes FGF9 stimulation resulted in an increased number of Sertoli cells (2503 vs. 3872 SOX9^+^ cells per mm^2^, *P* < 0.05). In fetal testes, inhibition of FGFR signalling by SU5402 treatment altered seminiferous cord morphology and reduced the AMH expression as well as the number of SOX9-positive Sertoli cells (2503 vs. 1561 SOX9^+^ cells per mm^2^, *P* < 0.05). In interstitial cells, reduced expression of COUP-TFII and increased expression of CYP11A1 and CYP17A1 in fetal Leydig cells was observed, although there were no subsequent changes in steroidogenesis.

**LARGE SCALE DATA:**

N/A

**LIMITATIONS, REASONS FOR CAUTION:**

*Ex vivo* culture may not replicate all aspects of fetal gonadal development and function *in vivo*. Although the effects of FGF9 were studied in *ex vivo* culture experiments, there is no direct evidence that FGF9 acts *in vivo* during human fetal gonadogenesis. The FGFR inhibitor (SU5402) used in this study is not specific to FGFR2 but inhibits all FGF receptors and off-target effects on unrelated tyrosine kinases should be considered.

**WIDER IMPLICATIONS OF THE FINDINGS:**

The findings of this study suggest that dysregulation of FGFR-mediated signalling may affect both testicular and ovarian development, in particular impacting the fetal germ cell populations in both sexes.

**STUDY FUNDING/COMPETING INTEREST(S):**

This work was supported in part by an ESPE Research Fellowship, sponsored by Novo Nordisk A/S to A.JØ. Additional funding was obtained from the Erichsen Family Fund (A.JØ.), the Aase and Ejnar Danielsens Fund (A.JØ.), the Danish Government’s support for the EDMaRC programme (A.JU.) and a Wellcome Trust Intermediate Clinical Fellowship (R.T.M., Grant no. 098522). The Medical Research Council (MRC) Centre for Reproductive Health (R.T.M.) is supported by an MRC Centre Grant (MR/N022556/1). The authors have no conflict of interest to disclose.

## Introduction

Development of ovaries or testes from a bipotential fetal gonad is a fundamental aspect of embryogenesis. This sex-specific differentiation involves a complex signalling cascade that directs gonad development based on cues from the somatic niche, resulting ultimately in the development of testes or ovaries (reviewed in Rotgers et al., 2018). Testicular differentiation is triggered by expression of SRY in pre-Sertoli cells, which in human fetal development is initiated from around 5–6 gestational weeks (GWs) (Berta et al., 1990; Sinclair et al., 1990). Subsequently, SRY triggers the expression of SOX9 and other male-promoting factors including FGF9 and PGD_2_ (Hanley et al., 2000; Ostrer et al., 2007), which have so far mainly been characterized in mice. Together, these factors promote early events relating to normal testis development, including regulation of somatic cell lineage differentiation and commitment of germ cells to the male developmental program, as well as inhibition of female pathway factors (reviewed in Windley and Wilhelm, 2015; Rotgers et al., 2018; Mäkelä et al., 2018). In humans, the initial testicular differentiation is distinguishable from 7–8 GWs when the gonocytes become surrounded by Sertoli cells and are enclosed within the forming seminiferous cords (Ostrer et al., 2007). At this stage, the fetal testis undergoes substantial reorganization directed by chemotactic signals produced by the Sertoli cells to establish the seminiferous cords and the interstitial compartment. The somatic niche ensures optimal support of the fetal gonocytes, which at this developmental time point are proliferating and actively prevented from prematurely entering meiosis (reviewed in Jørgensen and Rajpert-De Meyts, 2014). Human fetal gonocytes are characterized by expression of pluripotency markers, which are expressed until the gonocytes differentiate to pre-spermatogonia in an asynchronous manner starting towards the end of the first trimester (Mitchell et al., 2008).

Organogenesis of the fetal ovary is less well understood, especially in humans, but upon initiation of ovarian differentiation, expression of WNT4/RSPO1/β-catenin is stabilized. In human fetal gonads, expression of WNT4 is similar in males and females with no temporal fluctuation, whereas RSPO1 expression is ovary-specific (Tomaseli et al., 2011; Mamsen et al., 2017). Following initiation of the female fate by the WNT4/RSPO1/β-catenin pathway, granulosa cell fate is enforced by expression of FOXL2 (Ottolenghi et al., 2005; Uhlenhaut et al., 2009), which is distinguishable from around GW 8 in human ovaries (Jørgensen et al., 2015). The interstitial cell population in human fetal ovaries is characterized by expression of COUP-TFII with no co-expression between FOXL2-positive granulosa cells and COUP-TFII-positive stromal cells (Bashamboo et al., 2018). The oogonia are highly proliferative during first trimester and already at GW 9 there are approximately eight times more oogonia present in the ovaries compared to gonocytes in the testes (Bendsen et al., 2003; Bendsen et al., 2006). Another key event in early fetal ovary development is the initiation of meiosis, which starts from around 10–12 GWs in the human ovary and continues in an asynchronous manner, resulting in simultaneous presence of proliferating oogonia located in the periphery of the ovary and meiotic oocytes present deeper within the medulla (Anderson et al., 2007; Frydman et al., 2017). The initiation of meiosis is at least in part triggered by retinoic acid (Le Bouffant et al., 2010; Jørgensen et al., 2015), which stimulates the upregulation of the pre-meiosis marker STRA8 and subsequently the meiotic signalling cascade that includes SCP3, SPO11 and DMC1, coinciding with downregulation of pluripotency factor expression (Houmard et al., 2009; Le Bouffant et al., 2010; Childs et al., 2011; Jørgensen et al., 2012; Gkountela et al., 2013).

In mice, FGF9/FGFR2 is an important signalling pathway in the promotion of testis development that is activated just downstream of SRY and SOX9. Deletion of either FGF9 or FGFR2 in mice results in male-to-female sex reversal (Colvin et al., 2001; DiNapoli et al., 2006; Kim et al., 2007). Sex reversal has also been reported in a 46,XY patient with a FGFR2 mutation (Bagheri-Fam et al., 2015). The similar gonadal phenotype in knock-out (KO) models of FGF9 and FGFR2 suggests that FGF9 mediates its effects through the FGFR2 receptor during fetal testis development. Interestingly, in addition to its initially described role in promoting the male pathway, FGF9/FGFR2 signalling suppresses *Wnt4* expression in somatic cells, with initiation of *Wnt4* expression observed in *Fgf9*^–/–^ fetal testes (Kim et al., 2006). Accordingly, more recent results from double KO models (*Fgf9*/*Wnt4* and *Fgfr2*/*Wnt4*) suggest that the primary role of FGF9/FGFR2 signalling in fetal testis development is to ensure repression of the ovary-promoting gene *Wnt4* (Jameson et al., 2012). However, FGF9/FGFR2 signalling in mice is also involved in germ cell survival in fetal testes (DiNapoli et al., 2006), as well as in the promotion of male pathway genes and inhibition of meiotic initiation in mouse fetal gonads (Barrios et al., 2010; Bowles et al., 2010; Gustin et al., 2016). Interestingly, a recent study demonstrated that FGF9 signalling is active in human fetal ovaries and that FGF9 plays a role in the prevention of meiotic entry (Frydman et al., 2017). However, the role of FGF9/FGFR2 signalling in human fetal gonad development, especially in the testis, has not yet been characterized in detail. Therefore, the present study aimed to investigate the role of FGF9 signalling in both human fetal testis and ovary development by stimulating FGF9 signalling and inhibiting FGFR signalling in an established *ex vivo* culture model.

## Materials and Methods

### Collection of human fetal gonads and ethical approval

Human fetal gonads were isolated from material available following elective termination of pregnancy during the first trimester at the Department of Gynaecology at Copenhagen University Hospital (Rigshospitalet) and Hvidovre Hospital, Denmark. The regional ethics committee approved this study (permit number H-1-2012-007, including amendments 48801, 50662, 55184 and 64377) and women gave their informed written and oral consent. None of the terminations were for reasons of pathology of pregnancy or fetal abnormality. The embryos/fetuses included in this study were between 7 and 12 GWs, with fetal age determined by scanning crown-rump length and by evaluation of foot length (Evtouchenko et al., 1996). The fetuses were dissected in ice cold PBS and the isolated fetal gonads were immediately set up in *ex vivo* cultures. Gonad tissue from 44 male and 33 female fetuses was used for the *ex vivo* culture experiments followed by formalin fixation. Moreover, 12 fetal testis and 14 fetal ovary samples were immediately snap-frozen and stored at −80°C until further analysis by qRT-PCR. In total, this corresponded to 103 gonadal tissue samples. Some of the samples used for qRT-PCR analysis have also been used for gene expression analysis in a previous study (Jørgensen et al., 2018).

### *Ex vivo* gonad tissue culture

Human fetal gonads were cultured *ex vivo* in hanging drops as described previously (Jørgensen et al. 2015, 2018), with a few modifications. All gonads were initially divided into ~1 mm^3^ fragments prior to culture set up with at least one piece from each embryo/fetus used as vehicle control. The tissue was cultured in 40 μl medium for 14 days. Medium composition was: MEMα medium supplemented with 1× MEM non-essential amino acids, 2 mM sodium pyruvate, 2 mM L-glutamine, 1 × insulin, transferrin and selenium (ITS) supplement, (Sigma-Aldrich), 1 × penicillin/streptomycin, 10% fetal bovine serum. All cell media and supplements were from Gibco (Nærum, Denmark), except ITS (Sigma-Aldrich, Brøndby, Denmark). Fragments of gonads were cultured at 37°C under 5% CO_2_ with complete medium change every 48 h. Media from each tissue fragment was collected and pooled throughout the experimental period. To manipulate FGF signalling, fetal gonads were cultured in medium containing either 25 ng/ml recombinant human FGF9 (Sigma # SRP3040) or 10 μM SU5402 (FGFR tyrosine kinase inhibitor, Calbiochem #572630). FGF9 was dissolved in PBS with 0.1% BSA and SU5402 was dissolved in dimethyl sulfoxide vehicle (DMSO, 0.1%) (Sigma-Aldrich).

### Quantitative RT-PCR

Quantitative RT-PCR was conducted using the Mx300P platform (Stratagene, Cedar Creek, TX, USA), as previously described (Jørgensen et al., 2018). In brief, total RNA was extracted from frozen specimens and isolated with NucleoSpin RNA II purification kit, as described by the manufacturer (Macherey-Nagel, Düren, Germany). cDNA was synthesized using a dT20 primer and random hexamers. Gene expression was examined using specific primers (Table I) that were designed to span intron-exon boundaries and all amplicons were sequenced to verify specificity of primers (Eurofins Genomics, Ebersberg, Germany). Standard curve analysis and efficiency of amplification was established for all primers. Quantitative RT-PCR analysis was measured in technical triplicates using Stratagene Mx300P system with SYBR Green QPCR Master Mix (Stratagene, La Jolla, USA). PCR conditions were: 95°C 1 min + (95°C 30 s + 60°C 1 min + 72°C 1 min) × 40 + 95°C 1 min + 55°C 30 s + 95°C 30 s. Changes in gene expression were quantified using the 2^−∆∆Ct^ method. Expression levels were normalized to *RPS29* expression and calculated as a ratio relative to testis samples from 7–9 GWs, which were set to 1.

**Table I:** **Primer sequences**.

**Gene**	**Forward primer 5′-3′**	**Reverse primer 5′-3′**	**Amplicon size**	**GenBank Accession no.**
*FGF9*	CTACCTCGGGATGAATGAGAA	TTTCTGGTGCCGTTTAGTCCT	208 bp	NM_002010
*FGFR2*	GGCTTCCAGTCAAGTGGAT	TTCGTTGGTGCAGTTGGCT	203 bp	NM_000141
*OCT4 (POU5F1)*	TACTCCTCGGTCCCTTTCC	CAAAAACCCTGGCACAAACT	166 bp	NM_002701
*NR2F2*	TCCTGTTCACCTCAGATGCC	CTTTCCGAATCTCGTCGGCT	131 bp	NM_021005
*SOX9*	AGTACCCGCACTTGCACAAC	TCTCGCTCTCGTTCAGAAGTCT	75 bp	NM_000346
*CYP17A1*	GAGTTTGCTGTGGACAAGGG	CGCTGGATTCAAGAAACGCT	117 bp	NM_000102
*RPS29*	CGCTCTTGTCGTGTCTGTTCA	CCTTCGCGTACTGACGGAAA	91 bp	NM_001032

### Immunohistochemistry

Gonadal tissue was fixed in formalin immediately after the end of the *ex vivo* culture period. The fixed gonads were dehydrated, paraffin-embedded and sectioned (4 μm) using standard procedures. Immunohistochemistry (IHC) was conducted as previously described for formalin-fixed samples (Jørgensen et al., 2012). In brief, antigen retrieval was accomplished by microwaving the sections for 15 min in retrieval buffer. Sections were then incubated with 2% non-immune goat serum (Zymed Histostain kit, San Francisco, CA, USA) or 0.5% milk powder diluted in tris buffered saline (TBS) to minimize cross-reactivity. Primary antibodies, dilutions and retrieval buffers are listed in Table II. After 16 h of incubation at 4^o^C and 1 h at room temperature, the sections were incubated with biotinylated goat anti-rabbit IgG (Zymed Histostain kit) or biotinylated goat anti-mouse IgG, before a peroxidase-conjugated streptavidin complex (Zymed Histostain kit) was used as a tertiary layer. Visualisation was performed with amino ethyl carbasole (AEC) (NOVEX) yielding a red colour and sections were counterstained with Mayer’s haematoxylin. For all antibodies used, a positive control sample (tissue/cells known to express the studied protein) was included in the optimization of the antibody (Table II). Additionally, negative controls were included and processed with the primary antibody replaced by the dilution buffer alone. None of the negative control sections showed staining.

**Table II:** **Antibody dilutions, retrieval buffer and details**.

**Antibody**	**Dilution**	**Retrieval buffer**	**Company**	**Cat. Number**	**Positive control (tissue, cell type)**
OCT4	1:50	TEG	Santa Cruz	Sc-5279	Adult testis, GCNIS cells
NANOG	1:50	Citrate	R&D Systems	AF-1997	Adult testis, GCNIS cells
AP2γ	1:50	Urea	Santa Cruz	Sc-12762	Adult testis, GCNIS cells
MAGE-A4	1:250	TEG	Non-commercial	Gift from Prof. Spagnoli	Adult testis, Spermatogonia
AMH	1:400	Citrate	Santa Cruz	Sc-6886	Fetal testis, Sertoli cells
SOX9	1:400	Citrate	Millipore	AB5535	Adult testis, Sertoli cells
cPARP	1:75	Citrate	Cell Signaling	5625	Adult testis, nuclease treated
BrdU	1:100	Citrate	Dako	M0744	Adult testis culture, BrdU-treated
COUP-TFII	1:50	Citrate	Perseus Proteomics	PP-H7147-60	Adult testis, Interstitial cells
CYP11A1	1:250	TEG	Sigma	HPA016436	Adult testis, Leydig cells
CYP17A1	1:200	Citrate	Abcam	Ab134910	Adult testis, Leydig cells
3β-HSD	1:6000	Citrate	Non-commercial	Gift from Prof. Mason	Adult testis, Leydig cells
γH2AX	1:800	TEG	Abcam	Ab26350	Adult testis, Spermatocytes
SCP3	1:800	TEG	Novus	NB300-232	Adult testis, Spermatocytes
FOXL2	1:75	Citrate	Non-commercial	Gift from Dr. Wilhelm	Fetal ovary, Granulosa cells
WNT4	1:200	Citrate	Abcam	Ab-91226	Fetal ovary, Granulosa cells
FGF9	1:100	Citrate	Santa Cruz	Sc-7876	Fetal testis, Germ cells and Sertoli cells^*^

For all antibodies, antigen retrieval was conducted by microwaving sections in the indicated retrieval buffer. Citrate buffer: 10 mM, pH 6.0; TEG buffer: 10 mM Tris, 0.5 mM EGTA, pH 9.0; Urea buffer: 5% w/v carbamide, pH 5.5. ^*^As previously reported by Ostrer et al. (2007) using the same antibody.

### Immunofluorescence

Immunofluorescence (IF) was conducted as described in detail previously (Jørgensen et al., 2018). In brief, sections (4 μm) were dewaxed and rehydrated using standard procedures, followed by heat-induced antigen retrieval (pressure cooker) in 0.01 M citrate buffer (pH 6) and subsequent peroxidase block in 3% (v/v) H_2_O_2_ in methanol for 30 min. Sections were blocked in normal chicken serum (NCS; Biosera, Ringmer, UK) diluted 1:5 in TBS containing 5% (w/v) BSA. The primary antibodies OCT4 (Santa Cruz, sc-5279) and FGF9 (Santa Cruz, sc-7876) were both diluted 1:100, while secondary peroxidase-conjugated chicken anti-mouse and chicken anti-rabbit, respectively, were diluted 1:200. Following incubation with the appropriate secondary antibody, slides were incubated with Tyr-Cy3 and Tyr-Fluorescein (Perkin Elmer-TSA-Plus Cyanine3 System; Perkin Elmer Life Sciences, Boston, MA, USA) according to the manufacturer’s instructions. Sections were counterstained with DAPI (Sigma) diluted 1:500 in TBS for 10 min. Finally, slides were mounted with Permafluor (Thermo Scientific, UK) and fluorescent images were captured using an Olympus BX61 microscope (Olympus).

### BrdU incorporation

BrdU incorporation was used to determine the presence of proliferating germ cells just prior to the end of the *ex vivo* culture period, as previously described (Jørgensen et al., 2014). In brief, BrdU-labelling reagent (Life Technologies, Nærum, Denmark) was diluted 1:100 in culture medium and tissue fragments were placed in BrdU-containing media for 6 h. Tissue pieces were then washed twice in PBS for 5 min each, followed by fixation and paraffin embedding as described above. BrdU was visualized by IHC using a BrdU antibody (Table II) as described in the IHC section, with the positively stained cells considered as proliferating.

### Quantification of stained cells

To evaluate the IHC staining, the number of stained cells was quantified per area of tissue using entire tissue sections. The area was calculated using the NDPview software (Hamamatsu Photonics, Herrsching am Ammersee, Germany). Gonocytes and oogonia were identified based on OCT4 staining; proliferating cells were identified by BrdU incorporation; apoptotic cells were identified by cPARP staining; meiotic germ cells were identified by γH2AX staining (detects double-strand breaks and therefore potentially also apoptotic cells); and Sertoli cells were identified by SOX9 staining. For all quantifications, tissue samples from at least six embryos/fetuses were included.

### Steroid hormone measurements by LC-MS/MS

Steroid hormone levels in culture media following *ex vivo* culture were measured as described previously (Jørgensen et al., 2018). In brief, a sensitive isotope dilution TurboFlow-LC-MS/MS method was used for simultaneous quantification of dehydroepiandrosterone sulfate (DHEAS), Δ4-androstenedione, testosterone, 17α-hydroxyprogesterone, progesterone, 11-deoxycortisol, cortisol, cortisone, corticosterone and estrone 1-sulfate (E1-S) (Søeborg et al., 2017). The following modifications were applied: calibration curves were prepared in culture media; control samples were prepared by spiking with high and low concentration of steroids; and all collected media samples were diluted four times in culture media prior to analysis. A few samples were re-analysed for testosterone after additional sample dilution. Samples were analysed in 11 batches during summer/autumn of 2016. Each batch included standards for calibration curves, ~20 unknown samples, one blank and three pooled controls spiked with steroid standards at low and high levels. The day-to-day variation expressed as the relative standard deviation (RSD) was ≤6% for all analytes in both spike levels except for E1-S in a low spike level (22%). The recovery was >92% for all analytes in both spike levels. Additional details are listed in Table III.

**Table III:** **LC-MS/MS validation parameters for androgens and corticosteroids in cell media from fetal testis cultures**.

			Control Low			Control High		
	LOQ	Range	mean	RSD	recovery	mean	RSD	recovery
	(nM)	(nM)	(nM)	(%)	(%)	(nM)	(%)	(%)
Estrone 1-sulfate	0.026	LOQ-10	0.50	21.7	101	2.77	5.2	92
Cortisone	0.19	LOQ-112	1.90	5.5	95	23.4	4.8	94
Cortisol	1.9	LOQ-794	10.0	4.2	100	154	2.2	103
Dehydroepiandrosterone sulphate	19	LOQ-3000	48.3	8.8	97	614	8.7	102
Corticosterone	0.1	LOQ-144	1.99	4.3	100	28.7	3.3	96
11-deoxycortisol	0.017	LOQ-40	1.03	4.2	103	20.7	2.8	103
Δ4-androstenedione	0.042	LOQ-1746	19.1	3.1	95	285	2.5	95
Testosterone	0.012	LOQ-1732	9.69	4.4	97	724	2.5	97
17α-hydroxyprogesterone	0.1	LOQ-1513	26.3	4.5	105	438	3.8	109
Progesterone	0.036	LOQ-500	5.54	4.7	111	112	4.3	112

Limits of quantification (LOQ), range of calibration curves based on 10 standards and results of inter-day control materials (*n* = 32) at low and high levels from 11 batches. RSD: relative standard deviation.

### Statistical analysis

Statistical analysis was performed using GraphPad Prism Software. Data are presented as mean ± SEM. For cultured samples, Student’s paired (two-tailed) *t*-test was used. If samples were not paired, Student’s unpaired (two-tailed) *t*-test was used. Asterisks indicate statistical significance with ^*^P < 0.05, ^**^P < 0.01, ^***^P < 0.001. The number of replicates in each experimental setting and statistical significance are specified in each figure legend.

**Figure 1:**
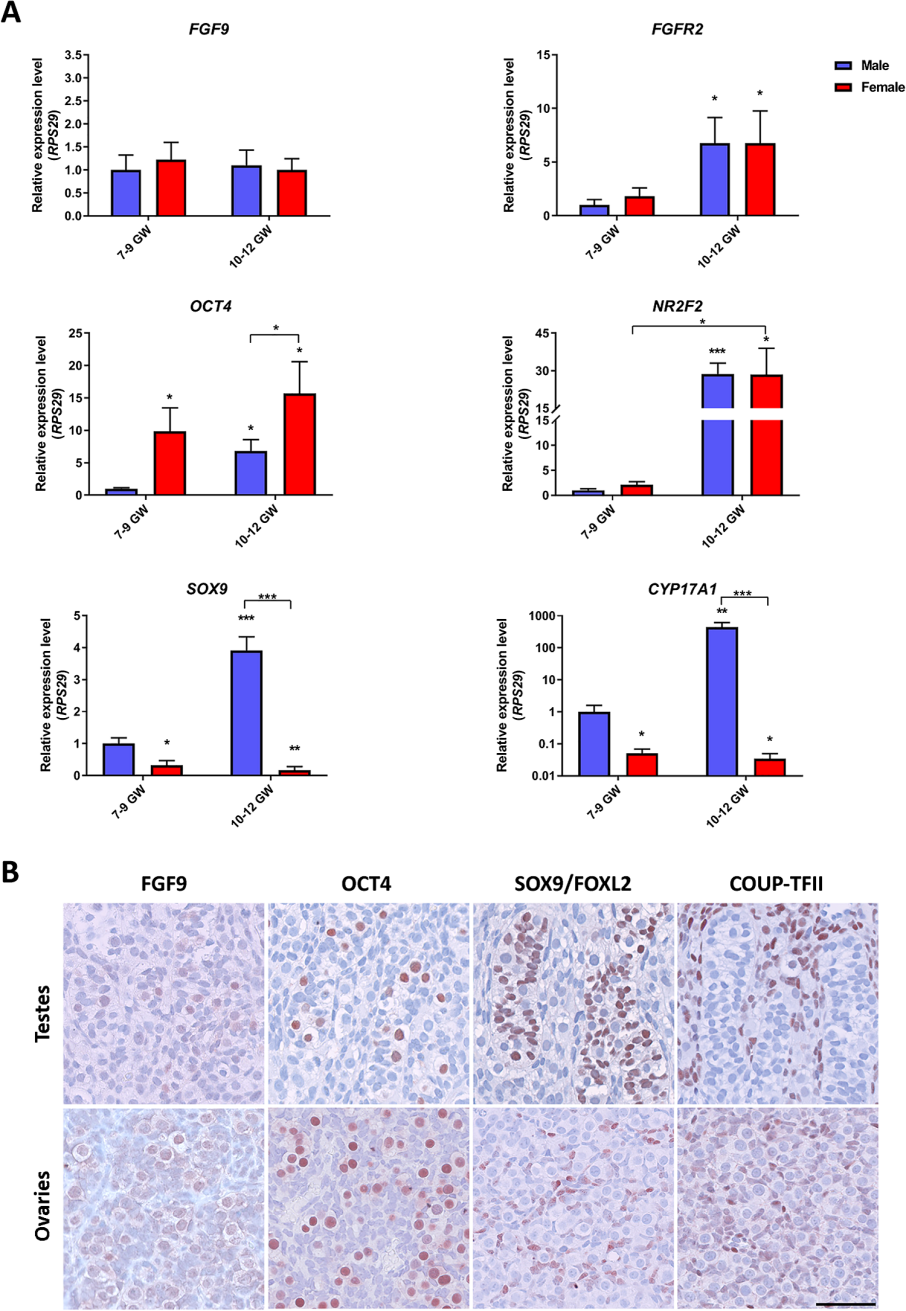
**Expression of FGF9, FGFR2 and fetal cell lineage markers in human fetal gonads.** (**A**) Transcriptional expression of *FGF9*, *FGFR2*, *OCT4 (POU5F1)*, *NR2F2*, *SOX9* and *CYP17A1* in ovary and testis samples from the first trimester. Gene expression is normalized to *RPS29* and expressed relative to testis samples from 7–9 GWs, which were set to a value of 1. Values represent mean ± SEM and for each age group *n* = 6–8. Significant difference compared to fetal testes 7–9 GWs, ^*^P < 0.05, ^**^P < 0.01, ^***^P < 0.001. GW: gestational week. (**B**) Immunohistochemical staining of FGF9, OCT4 (fetal germ cell marker), SOX9 (Sertoli cell marker, in fetal testis sample only), FOXL2 (granulosa cell marker, in fetal ovary sample only) and COUP-TFII (interstitial cell marker) in human ovary and testis samples from 10 GWs. Counterstaining with Mayer’s haematoxylin, scale bar corresponds to 50 μm.

**Figure 2:**
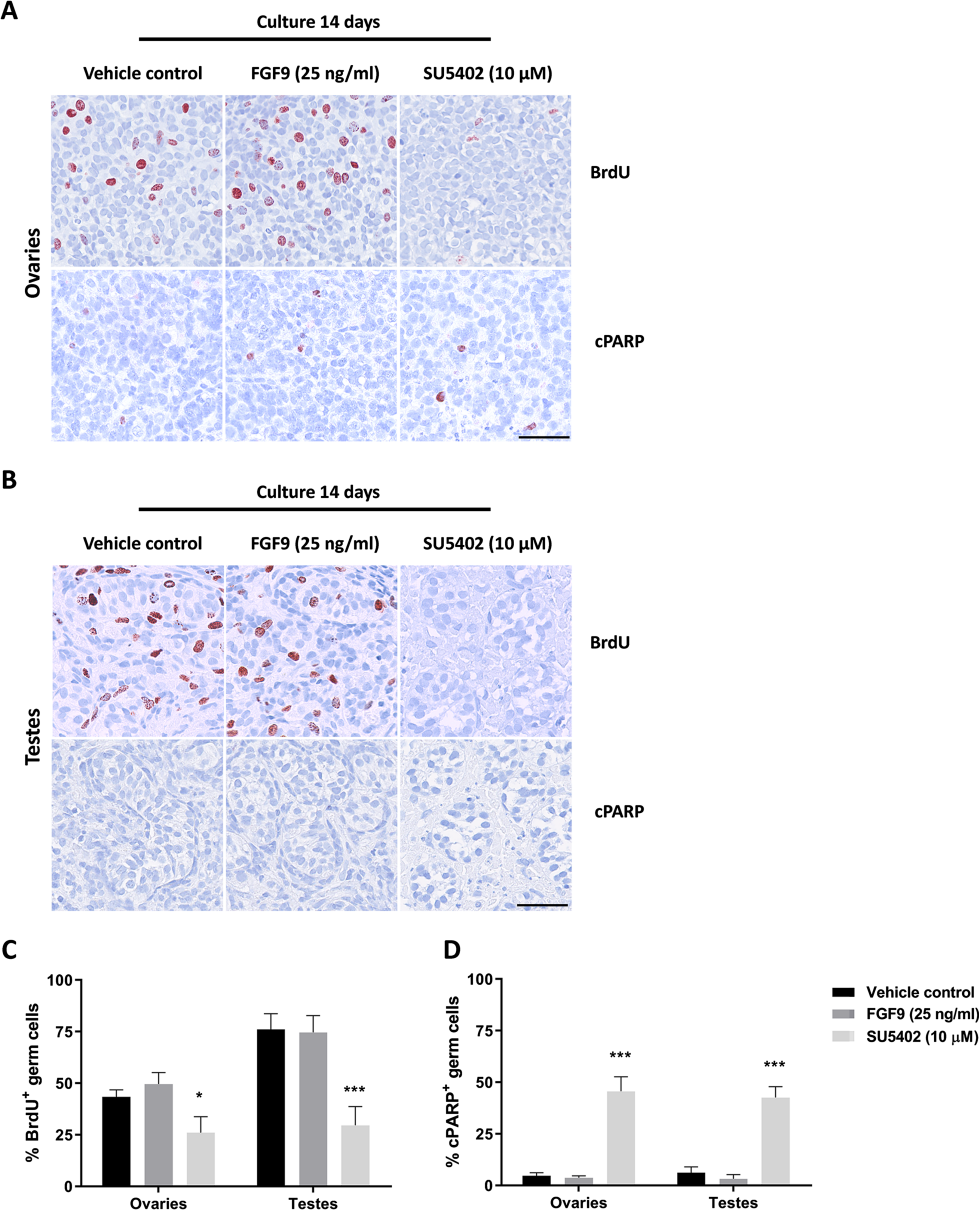
**Effects of manipulating FGF signalling on proliferation and apoptosis in human fetal gonads.** Effects on expression of markers of proliferation and apoptosis following FGF9 (25 ng/ml) and SU5402 (10 μM) treatment for 14 days in *ex vivo* cultures of fetal (**A**) ovaries and (**B**) testes. Immunohistochemical staining for the proliferation marker BrdU (which was added to culture media for the last 6 h of culture), and the apoptosis markers cleaved PARP (cPARP) was examined. Counterstaining with Mayer’s haematoxylin, scale bar corresponds to 50 μm. (**C**) Percentage of proliferating (BrdU^+^) germ cells after 14 days of culture in fetal ovaries and testes. Values represent mean ± SEM, with *n* = 10 (vehicle, ovaries), *n* = 7 (FGF9, ovaries), *n* = 6 (SU5402, ovaries), *n* = 9 (vehicle, testes), *n* = 8 (FGF9, testes), *n* = 7 (SU5402, testes). Significant difference compared to vehicle controls, ^*^P < 0.05, ^***^P < 0.001. (**D**) Percentage of apoptotic (cPARP^+^) germ cells after 14 days of culture in fetal ovaries and testes. Values represent mean ± SEM, with *n* = 10 (vehicle, ovaries), *n* = 8 (FGF9, ovaries), *n* = 6 (SU5402, ovaries), *n* = 10 (vehicle, testes), *n* = 8 (FGF9, testes), *n* = 7 (SU5402, testes). Significant difference compared vehicle controls, ^**^P < 0.001.

**Figure 3:**
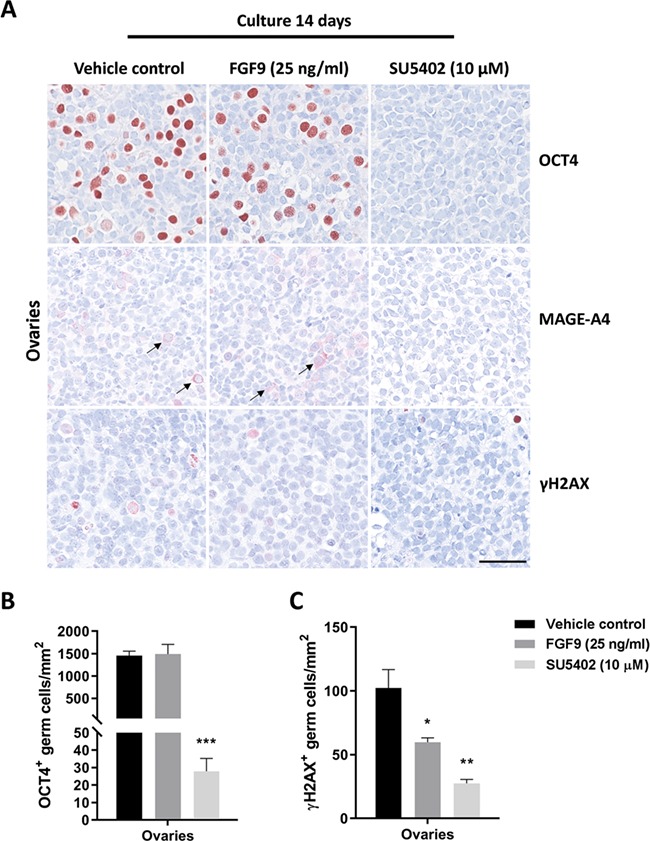
**Effects of manipulating FGF signalling on the expression of germ cell markers in human fetal ovaries.** (**A**) Expression pattern of germ cell markers OCT4 (oogonia), MAGE-A4 (subpopulation of oogonia transitioning into oocytes) and γH2AX (meiotic germ cells) in fetal ovary samples treated with FGF9 (25 ng/ml) and SU5402 (10 μM) for 14 days in *ex vivo* culture. Arrows indicate MAGE-A4^+^ cells. Counterstaining with Mayer’s haematoxylin, scale bar corresponds to 50 μm. (**B**) Quantification of germ cells determined as the number of OCT4^+^ gonocytes per mm^2^. Values represent mean ± SEM, with *n* = 14 (vehicle), *n* = 7 (FGF9) and *n* = 6 (SU5402). Significant difference compared to vehicle controls, ^***^P < 0.001. (**C**) Quantification of meiotic germ cells determined as the number of γH2AX^+^ germ cells per mm^2^. Values represent mean ± SEM, with *n* = 12 (vehicle), *n* = 7 (FGF9) and *n* = 6 (SU5402). Significant difference compared to vehicle controls, ^*^P < 0.05, ^**^P < 0.01.

## Results

### Expression of FGF9 and FGFR2 in human fetal testes and ovaries

To establish the presence of FGF9 and FGFR2 expression in human fetal testis and ovary samples during the first trimester, the transcriptional expression levels of *FGF9* and *FGFR2* were examined together with selected cell lineage markers *POU5F1* (OCT4), *NR2F2* (COUP-TFII), *SOX9* and *CYP17A1* (Fig. 1A). *FGF9* and *FGFR2* were expressed at similar levels in testis and ovary samples, but with a significant (*P* < 0.05) increase in the expression level of *FGFR2* from 7–9 GWs to 10–12 GWs in both sexes compared to testes at 7–9 GWs. The expression of *OCT4* was significantly (*P* < 0.05) higher in the ovaries compared to testes at both time intervals in the first trimester and there was a significantly (*P* < 0.05) higher *POU5F1* (*OCT4*) expression in testes at 10–12 GWs compared to 7–9 GWs. This most likely reflects the greater number of germ cells in fetal ovaries compared to fetal testes (Bendsen et al., 2003, 2006) as well as a high proliferation rate at the investigated developmental time point. The expression of *NR2F2* was similar between testis and ovary samples at 7–9 GWs, but with a significant increase (*P* < 0.001 and *P* < 0.05) in expression level in testes at 10–12 GWs, when compared to testes at 7–9 GWs. Moreover, the expression of *NR2F2* was significantly (*P* < 0.05) higher in ovaries at 10–12 GWs compared to 7–9 GWs. Expression levels of *SOX9* and *CYP17A1* were significantly higher in 7–9 GW testes compared to ovaries at 7–9 GWs (*P* < 0.05) and at 10–12 GWs (*P* < 0.01 and *P* < 0.05, respectively). Moreover, there was a significant increase (*P* < 0.001 and *P* < 0.01) in the expression levels of both *SOX9* and *CYP17A1* in testes at 10–12 GWs compared to testes at 7–9 GWs, respectively. Finally, expression of *SOX9* and *CYP17A1* was significantly higher (*P* < 0.001) in 10–12 GW testes compared to 10–12 GW ovaries. At the protein level, FGF9 was expressed in germ cells in both fetal testes and ovaries as well as in Sertoli cells, a sub-population of interstitial cells in testes and a sub-population of somatic cells in ovaries (Fig. 1B, Supplementary Fig. S1A). However, this expression pattern differs to some extent compared to a previous study that examined FGF9 expression in human fetal gonads using a different antibody (Frydman et al., 2017). Other cell lineage markers: OCT4 (germ cells), SOX9 (Sertoli cells), FOXL2 (granulosa cells) and COUP-TFII (interstitial cells) were expressed as expected (Fig. 1B).

### Effects on proliferation and apoptosis following manipulation of FGF signalling in human fetal gonad cultures

Next, the effect of manipulating FGF signalling in *ex vivo* cultures of human fetal testes and ovaries was examined by treatment with either recombinant FGF9 (25 ng/ml) or the FGFR-inhibitor SU5402 (10 μM) for 2 weeks. Effects on proliferation were examined based on BrdU incorporation, and apoptosis was detected based on expression of cleaved PARP (cPARP). In the ovaries, it was evident that treatment with the SU5402 inhibitor resulted in fewer BrdU^+^ cells, while no apparent effect of FGF9 stimulation was observed (Fig. 2A). Overall, only few cPARP^+^ cells were found in the ovary cultures regardless of treatment (Fig. 2A). In the testis cultures, treatment with SU5402 also reduced the number of BrdU^+^ cells, and again no apparent difference was observed between FGF9-treated samples and vehicle controls (Fig. 2B). Importantly, in vehicle control and FGF9-stimulated ovary and testis samples, both germ cells and somatic cells continued to proliferate during the culture period, indicating that the culture approach supports continued cell proliferation. Few cPARP^+^ cells were detected in testis cultures after 14 days of culture regardless of treatment, although it appeared that more cPARP^+^ cells were present in SU5402 treated testis samples and overall more cPARP^+^ cells were found in fetal ovary compared to testis samples. The observation that treatment with SU5402 reduced germ cell proliferation was confirmed after quantification of the percentage of BrdU^+^ germ cells, which was significantly reduced (*P* < 0.05 and *P* < 0.001) in ovaries and testes, respectively (Fig. 2C). No effect of FGF9 stimulation on proliferation of germ cells was found in either fetal ovaries or testes (Fig. 2C). Additionally, quantification of the percentage of cPARP^+^ germ cells demonstrated that treatment with SU5402 significantly increased (*P* < 0.001) the number of apoptotic cells in both ovaries and testes (Fig. 2D). In contrast, stimulation of FGF9 signalling did not affect the percentage of cPARP^+^ germ cells compared to vehicle controls in either ovaries or testes.

**Figure 4:**
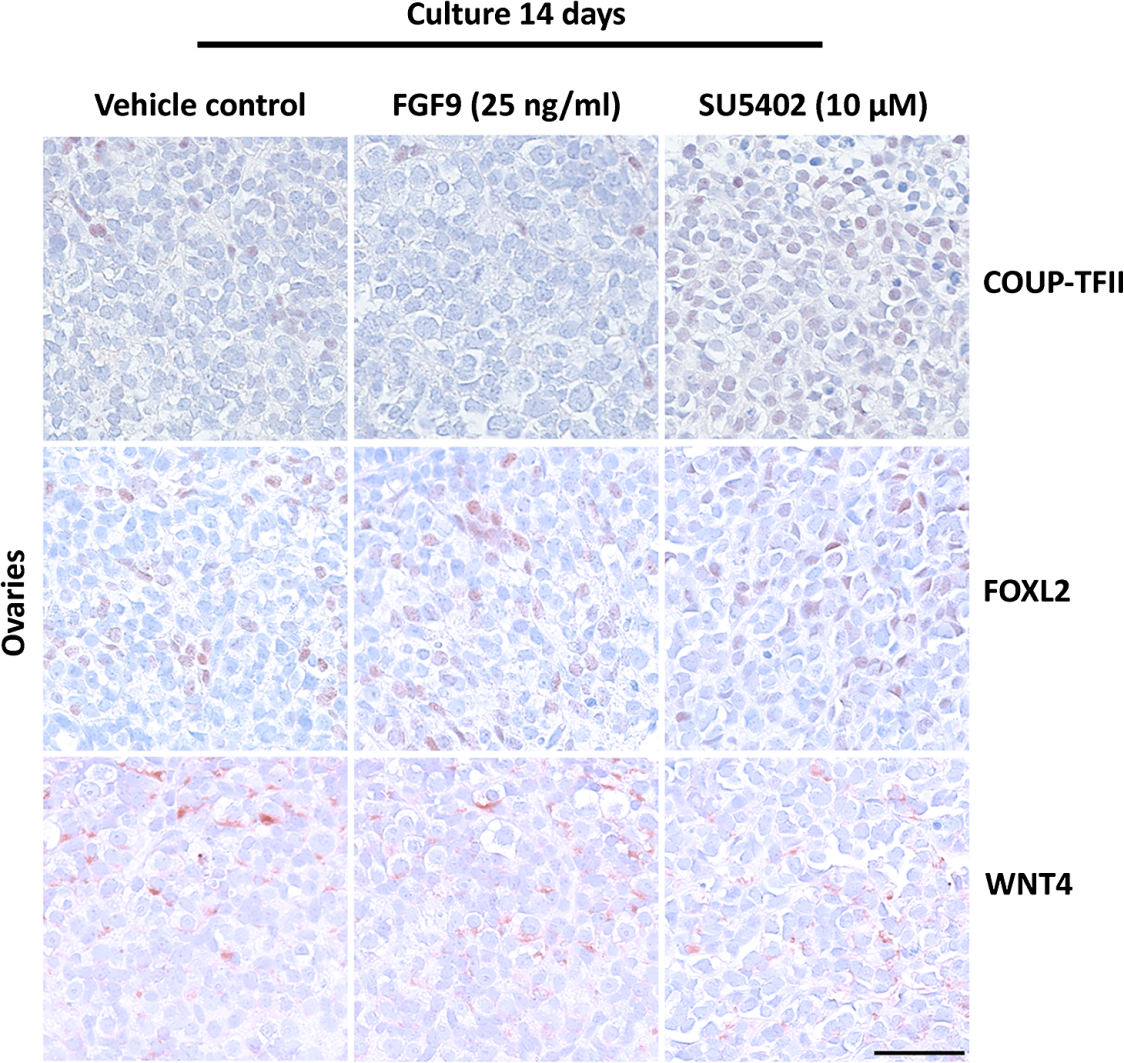
**Effects of manipulating FGF signalling on expression of somatic cell markers in human fetal ovaries.** Expression pattern of somatic cell markers COUP-TFII (interstitial cells), FOXL2 (granulosa cells) and WNT4 (primarily in granulosa cells) in fetal ovary samples treated with FGF9 (25 ng/ml) and SU5402 (10 μM) for 14 days in *ex vivo* culture. Counterstaining with Mayer’s haematoxylin, scale bar corresponds to 50 μm.

### Effects on expression of cell lineage markers in human fetal ovary cultures following manipulation of FGF signalling

Stimulation of FGF9 signalling did not affect the overall expression pattern of OCT4 in oogonia compared to vehicle controls, but after treatment with the SU5402 inhibitor, expression of OCT4 was almost completely abolished (Fig. 3A). This observation was confirmed after quantification of OCT4^+^ cells per area, where a significantly lower (1456 vs. 28, *P* < 0.001) number of oogonia (OCT4^+^) were found following SU5402 treatment (Fig. 3B). To distinguish whether this effect was due to specific downregulation of pluripotency factors, differentiation or loss of the oogonia population, additional markers were examined. Examination of the pluripotency markers AP2γ and NANOG resulted in a similar finding as for OCT4 with almost no positive cells detected (data not shown). Therefore, expression of MAGE-A4 (marker for differentiating oogonia) was examined to establish whether FGF9 and/or SU5402 treatment promoted differentiation. However, few MAGE-A4^+^ cells were detected, with no difference between the vehicle controls and samples treated with FGF9 or SU5402 (Fig. 3A). To establish whether manipulation of FGF signalling affected initiation of meiosis in germ cells, expression of the meiosis marker γH2AX was examined. γH2AX^+^ cells were present in cultured fetal ovaries regardless of treatment (Fig. 3A), but since γH2AX marks double-strand breaks, the positive cells may be undergoing apoptosis and therefore morphological assessment is required to ensure that γH2AX^+^ cells are indeed meiotic. Quantification of meiotic cells demonstrated that treatment with FGF9 and SU5402 both resulted in a significantly lower (*P* < 0.05 and *P* < 0.01, respectively) number of γH2AX^+^ cells per area (Fig. 3C). However, the reduced number of γH2AX^+^ germ cells in SU5402-treated ovaries is most likely a consequence of the very low number of germ cells remaining in these samples and not SU5402-mediated inhibition of meiotic entry. To assess the effects of SU5402 treatment on the somatic niche in fetal ovaries, somatic cell lineage markers, COUP-TFII (stromal/interstitial cells), FOXL2 (granulosa cells) and WNT4 (granulosa cells), were examined by IHC in fetal ovary samples following treatment with FGF9 and SU5402. However, no apparent difference in the expression pattern of any of these somatic cell lineage markers was observed compared to vehicle controls (Fig. 4).

### Effects on expression of cell lineage markers in human fetal testis cultures following manipulation of FGF signalling

**Figure 5:**
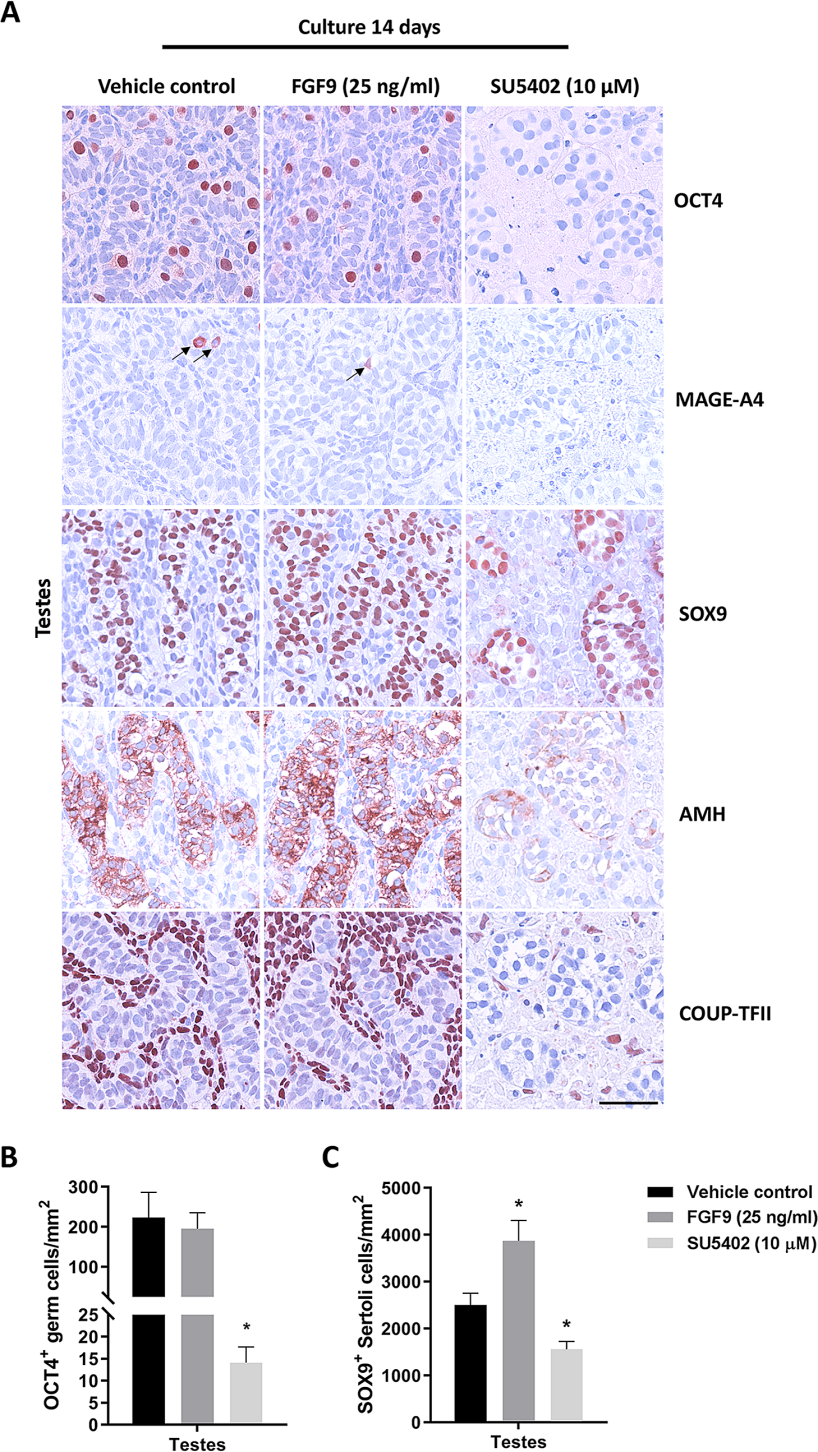
**Effects of manipulating FGF signalling on expression of germ cell and somatic cell markers in human fetal testes.** (**A**) Expression pattern of germ cell markers OCT4 (gonocytes), MAGE-A4 (pre-spermatogonia) and somatic cell markers SOX9 (Sertoli cells), AMH (Sertoli cells) and COUP-TFII (interstitial cells) in fetal testis samples treated with FGF9 (25 ng/ml) and SU5402 (10 μM) for 14 days in *ex vivo* culture. Arrows indicate MAGE-A4^+^ cells. Counterstaining with Mayer’s haematoxylin, scale bar corresponds to 50 μm. (**B**) Quantification of germ cells determined as the number of OCT4^+^ gonocytes per mm^2^. Values represent mean ± SEM, with *n* = 12 (vehicle), *n* = 8 (FGF9) and *n* = 7 (SU5402). Significant difference compared to vehicle controls, ^*^P < 0.05. (**C**) Quantification of Sertoli cells determined as the number of SOX9^+^ cells per mm^2^. Values represent mean ± SEM, with *n* = 10 (vehicle), *n* = 8 (FGF9) and *n* = 7 (SU5402). Significant difference compared to vehicle controls, ^*^P < 0.05.

Stimulation of FGF9 signalling did not appear to affect the expression of germ cell markers OCT4 and MAGE-A4, Sertoli cell markers SOX9 and AMH or the interstitial cell marker COUP-TFII (Fig. 5A). In contrast, treatment with SU5402 resulted in pronounced morphological alterations in the fetal testis tissue, with smaller and more rounded seminiferous cords. The cords contained Sertoli cells that appeared larger and were located closer to the basement membrane, while the interstitial area was increased compared to vehicle-treated samples (Fig. 5A). Moreover, in accordance with the results from the fetal ovary cultures, treatment with SU5402 in fetal testes resulted in almost complete loss of OCT4^+^ gonocytes (Fig. 5A). To examine whether this lack of OCT4^+^ germ cells was a consequence of premature differentiation to pre-spermatogonia (MAGE-A4^+^), specific downregulation of OCT4 expression or loss of the germ cell population, additional germ cell markers were included in the analysis. Since almost no pre-spermatogonia (MAGE-A4^+^) cells were detected (Fig. 5A) and no AP2γ^+^ or LIN28^+^ cells were found (data not shown), it appeared that the majority of the gonocyte population in fetal testis was lost as a consequence of SU5402 treatment. This was also evident after quantification of OCT4^+^ cells per area, which was significantly reduced (224 vs. 14 OCT4^+^ per mm^2^, *P* < 0.05) in SU5402-treated fetal testis samples (Fig. 5B). The SU5402 treatment also reduced the expression of somatic cell lineage markers AMH (Sertoli cells) and COUP-TFII (interstitial cells) (Fig. 5A), but did not promote trans-differentiation of Sertoli cells towards granulosa-like cells based on the lack of FOXL2 expression (data not shown). In contrast, treatment with SU5402 did not affect the expression of SOX9 in Sertoli cells, but the number of Sertoli cells appeared reduced (Fig. 5A). This was confirmed by quantifying the number of SOX9^+^ cells per area, which was significantly lower (2503 vs. 1561, *P* < 0.05) following treatment with SU5402 compared to vehicle controls (Fig. 5C). Interestingly, this quantification also demonstrated that stimulation of FGF9 signalling had the opposite effect and significantly increased (2503 vs. 3872, *P* < 0.05) the number of SOX9^+^ cells per area compared to vehicle controls, indicating involvement of FGF9 in Sertoli cell proliferation.

### Effects of manipulating FGF signalling on expression of Leydig cell markers and steroidogenesis in fetal testis cultures

**Figure 6:**
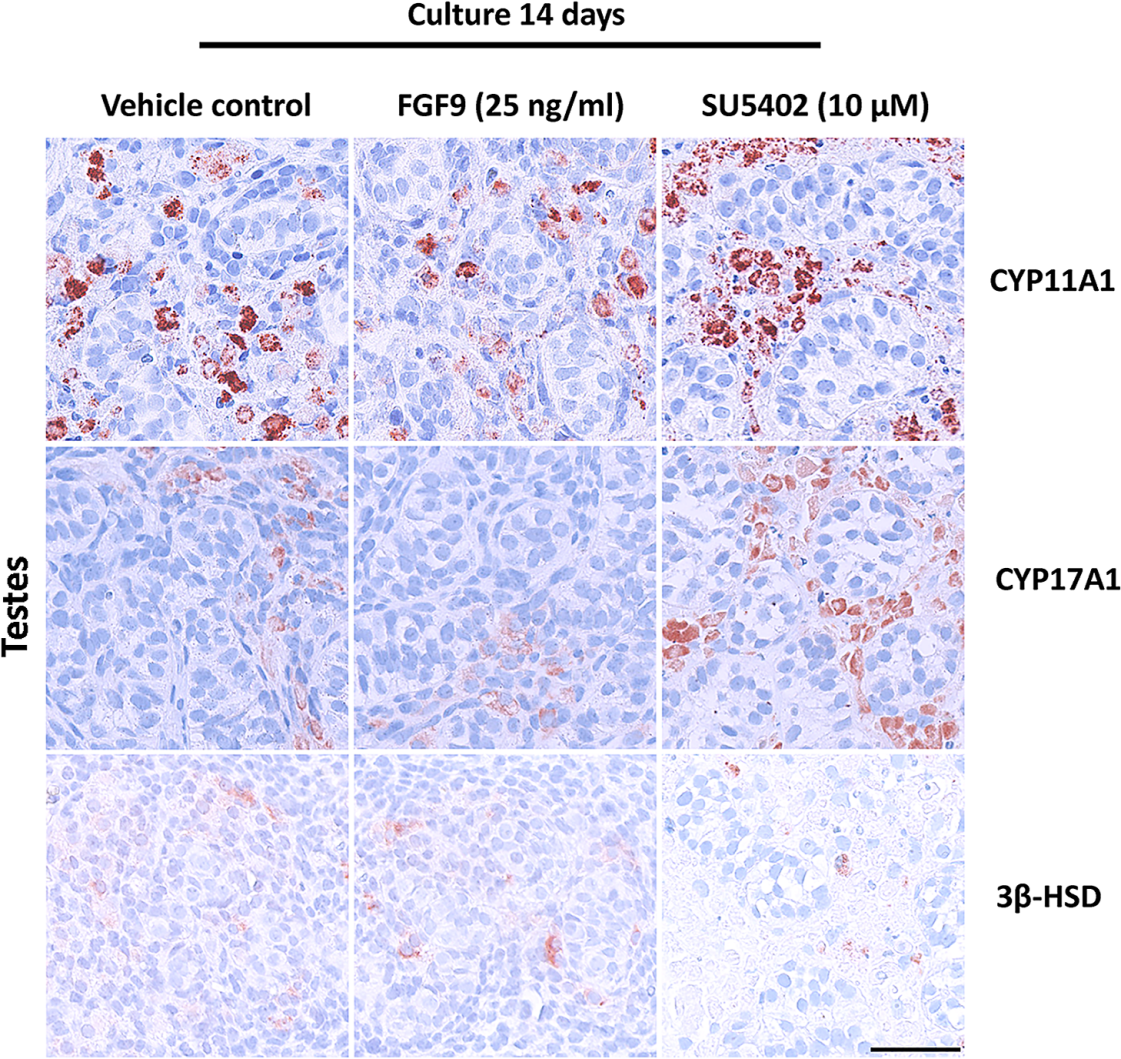
**Effects of manipulating FGF signalling on expression of Leydig cell markers in human fetal testes.** Expression pattern of fetal Leydig cell markers CYP11A1, CYP17A1 and 3β-HSD in fetal testis samples treated with FGF9 (25 ng/ml) and SU5402 (10 μM) for 14 days in *ex vivo* culture. Counterstaining with Mayer’s haematoxylin, scale bar corresponds to 50 μm.

**Figure 7:**
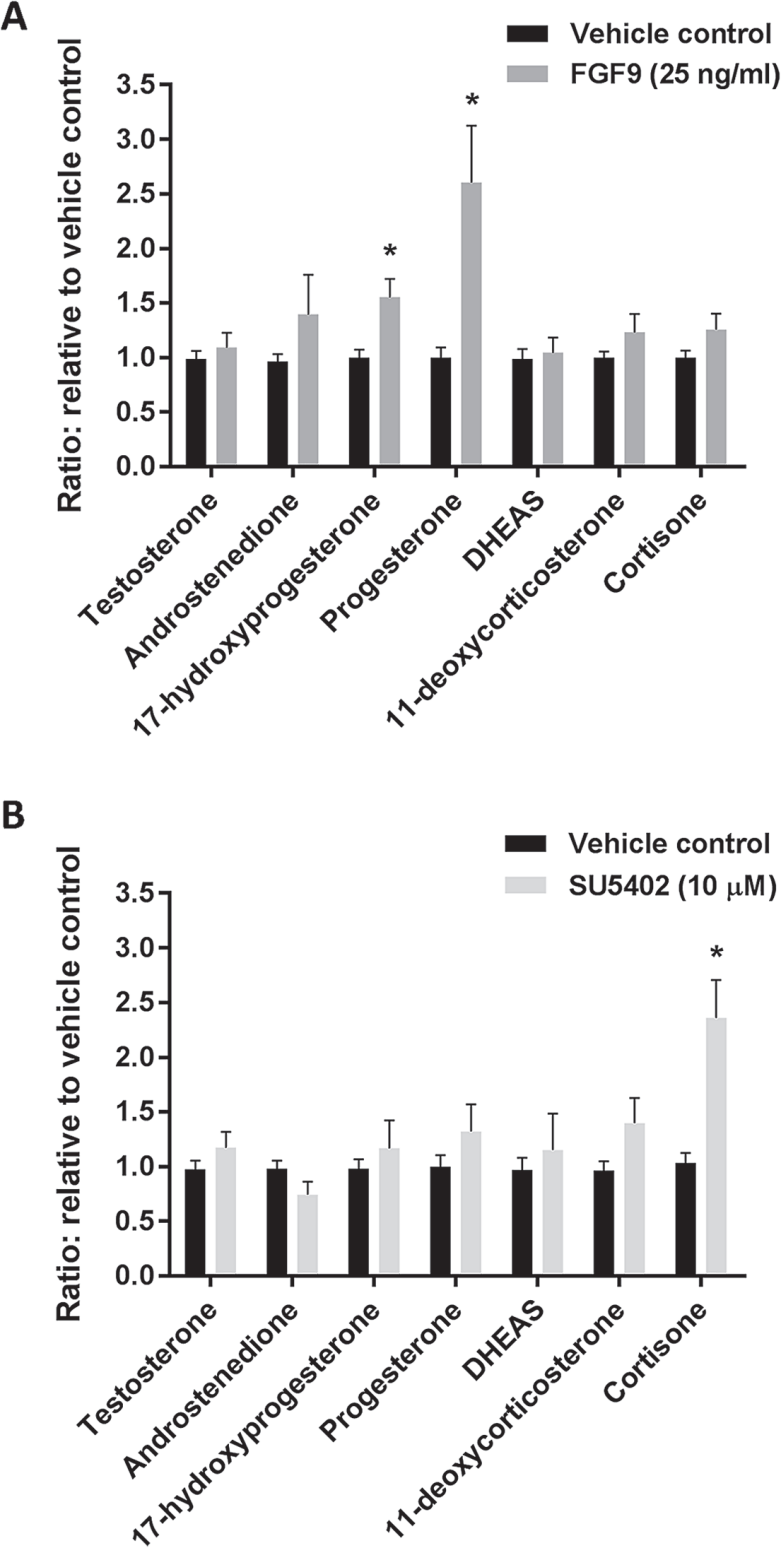
**Effects of manipulating FGF signalling on steroidogenesis in human fetal testes.** Quantification of steroid hormone metabolites produced in *ex vivo* cultures of fetal testis tissue and secreted to the media droplets. Medium was collected every 48 h throughout the 14 days culture period and was pooled for each individual tissue piece. Androgens were measured by LC-MS/MS and are shown as ratios compared to corresponding vehicle controls. (**A**) Effect of FGF9 (25 ng/ml) treatment for 14 days in *ex vivo* culture. Values represent mean ± SEM, with *n* = 16 (vehicle) and *n* = 16 (FGF9). Significant difference compared to vehicle control, ^*^P < 0.05. (**B**) Effect of SU5402 (10 μM) treatment for 14 days in *ex vivo* culture. Values represent mean ± SEM, with *n* = 12 (vehicle) and *n* = 12 (SU5402). Significant difference compared to vehicle control, ^*^P < 0.05.

Stimulation of FGF9 signalling did not result in any apparent effects on the expression of the Leydig cell markers CYP11A1, CYP17A1 and 3β-HSD (Fig. 6). In contrast, treatment with SU5402 resulted in an apparent increase in expression of CYP11A1 and CYP17A1, but did not appear to affect 3β-HSD expression (Fig. 6). To determine whether fetal Leydig cell function was altered following FGF9 and SU5402 treatment, steroidogenesis was evaluated by analysis of media collected from *ex vivo* cultures throughout the experimental period. FGF9 treatment resulted in significantly higher levels of 17-hydroxyprogesterone (ratio 1 vs. 1.5, *P* < 0.05) and progesterone (ratio 1 vs. 2.6, *P* < 0.05) (Fig. 7A), while SU5402 treatment did not result in alterations in steroidogenesis, except for a significantly higher level of cortisone (ratio 1 vs. 2.4, *P* < 0.05) (Fig. 7B).

## Discussion

During recent years, much progress has been made in the understanding of molecular mechanisms and signalling pathways underlying gonadal sex differentiation in mice. However, only a few studies have investigated these in human development mainly due to the difficulty in obtaining fetal tissue. In the present study, we investigated the involvement of FGF9, and signalling through FGFR, in human fetal gonad development, taking advantage of our established *ex vivo* culture model (Jørgensen et al., 2015, 2018). We demonstrated that following our attempt to inhibit FGFR signalling by treatment with SU5402, a severely reduced number of germ cells in both human fetal testes and ovaries was observed. In addition, inhibition of FGF signalling by use of SU5402 also affected the somatic niche in human fetal testes, including effects on testicular morphology and expression pattern of cell lineage markers, while stimulation of FGF9 signalling promoted Sertoli cell proliferation in testes and inhibited meiotic entry in ovaries. These findings demonstrate the potential importance of the FGF9 signalling pathway in human fetal gonad development.

Inhibition of FGFR signalling by SU5402 treatment severely impaired the germ cell population with almost complete loss of OCT4^+^ cells in gonads of both sexes, which was explained by both reduced germ cell proliferation and increased apoptosis. Despite the increase in percentage of apoptotic cells after 2 weeks in culture, it is likely that the majority of germ cells in SU5402-treated samples were lost at an earlier time point during the experimental period. Similar results were recently demonstrated following simultaneous inhibition of Nodal and Activin signalling in human fetal testes using the same experimental approach (Jørgensen et al., 2018). In contrast, stimulation of FGF9 signalling in human fetal gonads did not affect proliferation or apoptosis of the germ cells. These findings are partly in accordance with previous studies in *Fgf9* KO mice, in which germ cell loss (by apoptosis) occurred in fetal testes, but not in ovaries (DiNapoli et al., 2006). The observed reduction in germ cell number, resulting from both increased apoptosis and reduced proliferation following treatment with SU5402 in human fetal ovaries is in line with the observed expression of FGF9 in germ cells of human fetal gonads of both sexes, which was reported in the present study as well as in previous studies (Ostrer et al., 2007; Frydman et al., 2017). This suggests a role for this signalling pathway in germ cell maintenance in both human fetal testes and ovaries. In contrast, expression of *Fgf9*/FGF9 in mice becomes testis-specific upon initiation of gonadal sex differentiation (Colvin et al., 2001; Nef et al., 2005; Kim et al., 2006). Stimulation of FGF9 signalling in human fetal ovary cultures reduced the number of meiotic germ cells, which is in accordance with a previous study (Frydman et al., 2017). This was despite the use of a lower concentration of FGF9 (25 ng/ml) in the present study compared to 1 μM FGF9 used previously (Frydman et al. 2017). However, Frydman et al. (2017) also reported that inhibition of FGFR signalling (using a different early generation FGFR inhibitor, PD173074, 1 μM) increased the number of meiotic germ cells in fetal ovaries, which is in contrast to the present study where a reduced number of meiotic cells was found after FGFR inhibition (SU5402, 10 μM). We consider the result of the present study to most likely be a consequence of the overall loss of the germ cell population and not a direct effect on initiation of meiosis. In fetal mouse ovary cultures treatment with FGF9 resulted in repressed WNT4 expression (Kim et al., 2006; Jameson et al., 2012) as well as upregulation of pluripotency markers *Oct4* and *Sox2* and male-fate markers *Nanos2* and *Dnmt3l* (Bowles et al., 2010). In contrast to the studies in mice, the present study showed no effects on the number of OCT4^+^ cells or the expression of somatic cell lineage markers WNT4, FOXL2 and COUP-TFII after treatment with recombinant FGF9 or SU5402 in human fetal ovary cultures.

In contrast to the ovaries, manipulation of FGF signalling in fetal testes affected the somatic niche. Stimulation of FGF9 signalling resulted in an increased number of Sertoli cells, which is in line with previous studies in mice where FGF9 was shown to promote proliferation (and differentiation) of Sertoli cells (Schmahl et al., 2004). In accordance, the present study demonstrated that treatment with SU5402 reduced the number of SOX9^+^ cells and also the presence of proliferating Sertoli cells appeared to be reduced. These findings are also in line with mouse studies where a reduced Sertoli cell number was reported in *Fgf9* KO mice, which also had smaller testes and failed to develop seminiferous cords (Colvin et al., 2001; Schmahl et al., 2004). Interestingly, we observed that the human testis tissue fragments treated with SU5402 were clearly smaller compared to both vehicle controls and other treatments. Even though it was not possible to quantify the growth of the testis fragments in culture in the present study, this observation could indicate involvement of FGF9 signalling in mediating fetal testis growth also in humans. In the present study, treatment with SU5402 did not affect the formation or maintenance of seminiferous cords in human fetal testis cultures, despite our previous studies demonstrating pronounced effects on this end-point following inhibition of Nodal signalling (Jørgensen et al., 2018) and following treatment with exogenous retinoic acid (Jørgensen et al., 2015). Despite the presence of intact seminiferous cords in SU5402-treated human fetal testes in the present study, some effects were observed, including a rounder shape of tubules with Sertoli cells appearing larger and located closer to the basement membrane. Moreover, in human fetal testis cultures treated with SU5402, we observed reduced Sertoli cell expression of AMH, which is in accordance with results from *Fgfr2* KO mice (Bagheri-Fam et al., 2015) as well as a patient with 46,XY monosomy 10q (including the *FGFR2* gene) (Tosur et al., 2015). Interestingly, the patient had reduced serum AMH levels, but the testosterone levels were within the normal range (Tosur et al., 2015) and in the present study, we found that treatment with SU5402 had no overall effect on steroidogenesis, including the testosterone levels. This was to some extent in contrast to the apparent increase in the expression of steroidogenic enzymes CYP11A1 and CYP17A1 observed following SU5402 treatment. The stimulation of FGF9 signalling resulted in increased levels of 17-hydroxyprogesterone and progesterone in media from testis cultures. This finding could indicate increased 3β-HSD enzyme activity, despite the apparent lack of increased 3β-HSD protein expression compared to vehicle controls. In fetal mouse urogenital ridge cultures, treatment with SU5402 (5 μM) resulted in downregulation of male-promoting genes *Nanos2* and *Dnmt3l* (Bowles et al., 2010), which is in line with the present study where downregulation of AMH and COUP-TFII expression was observed in addition to the reduced number of SOX9^+^ cells. In the study by Wu et al. (2013), treatment of testis cultures with SU5402 (40 μM) resulted in upregulation of the female-promoting genes *Wnt4*, *Foxl2*, *Fst* and *Bmp2* (Wu et al., 2013). This is in contrast to the present study where expression of FOXL2 could not be detected in fetal testes treated with a lower dose of SU5402 (10 μM).

The FGFR-inhibitor (SU5402) selected for the present study has previously been used to investigate the effects of inhibiting FGF9 signalling in fetal mouse testis and ovary cultures, using concentrations ranging from 5 μM (Bowles et al., 2010; Spiller et al., 2012), 20 μM (Tian-Zhong et al., 2016) up to 40 μM (Wu et al., 2013). In a recent study comparing the ability of different FGFR tyrosine kinase (TK) inhibitors to rescue FGF2-mediated inhibition of rat chondrocyte proliferation, the lowest concentration of SU5402 needed to rescue this phenotype was determined to be 10 μM (Gudernova et al., 2016), which is also in keeping with a previous study using 10 μM SU5402 to induce human embryonic stem cell differentiation (Vallier et al., 2005). This suggests that the dose of 10 μM used in the present study is appropriate. However, the study by Gudernova et al. (2016) highlighted that all investigated FGFR inhibitors, including SU5402, targeted the activity of all FGFRs and exhibited significant off-target activity, which was especially pronounced for SU5402. Therefore, caution is warranted in the interpretation of results following SU5402-mediated FGFR-inhibition, since it is not possible to exclude that some of the observed effects are the results of simultaneous inhibition of other tyrosine kinases. Additionally, since the expression pattern of FGF9 in human fetal gonads to some extent differs between the IHC and IF stainings in the present study as well as when compared to previous studies (Ostrer et al., 2007; Frydman et al., 2017), this is a limitation of our study. We detected expression of FGF9 in germ cells in fetal testes and ovaries, which is in accordance with a previous study reporting expression in male germ cells using the same antibody (Ostrer et al., 2007). Furthermore, single cell RNA sequencing (scRNA-seq) data also indicated *FGF9* expression in germ cells from both sexes (Li et al., 2017, data shown in Supplementary Fig. 1B and C). In contrast, Frydman et al. (2017) did not report expression of FGF9 in germ cells in human fetal ovaries or testes using a different antibody. In the present study, expression of FGF9 was consistently detected in germ cells following IHC and IF, while IF also resulted in staining in several other cell types. Whether the reported expression of FGF9 in germ cells in human fetal gonads results from non-specific staining of the used antibody requires further investigation and therefore the expression pattern of FGF9 in the present study should be interpreted with caution.

**Figure 8:**
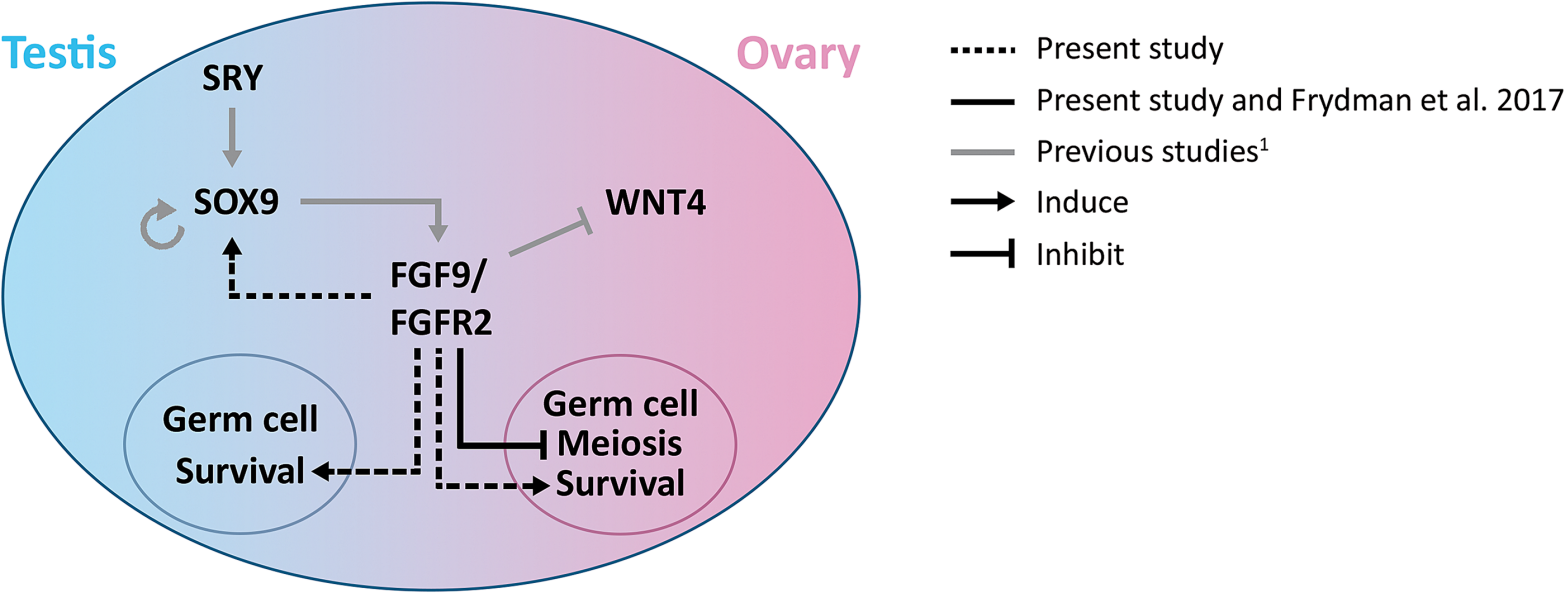
**Proposed involvement of FGF9/FGFR2 signalling in human fetal gonad development.** A model illustrating the interaction between key factors involved in early gonad development. SRY upregulates SOX9 in Sertoli cells of the developing testes resulting in the self-promoting expression of SOX9, which is followed by expression of additional testis-promoting factors, including FGF9. FGF9 signals through FGFR2 to inhibit the pro-ovarian factor WNT4 (and the RSPO1/β-catenin pathway). The present study confirmed the FGF9-mediated inhibition of meiotic entry in female germ cells previously demonstrated in human fetal ovaries (Frydman et al., 2017). Additionally, this study suggests the involvement of FGF9/FGFR2 in the expression of SOX9 in human fetal testes. Finally, the present study suggests involvement of FGF9/FGFR2 signalling in supporting germ cell survival in both sexes.^1^ Reviewed in Rotgers et al., 2018.

In conclusion, the present study demonstrated that pharmaceutical inhibition of FGFR signalling in human fetal gonads, by treatment with SU5402, had pronounced effects on survival of the germ cell population, resulting in a severely reduced number of gonocytes and oogonia in fetal testes and ovaries, respectively. In addition, inhibition of FGFR signalling affected the somatic niche in human fetal testes, with effects on testicular morphology and expression pattern of cell lineage markers. Stimulation of FGF9 signalling resulted in a higher number of Sertoli cells in testes, while in ovaries we observed FGF9-mediated inhibition of meiotic entry. The findings of this study are summarized in Figure 8 in the context of previous mouse and human studies. Together, our findings indicate that FGF9 signalling may be involved in the signalling cascade directing human gonadal differentiation and suggest that dysregulation of this pathway may have consequences for both testicular and ovarian development in humans.

## Supplementary Material

Suppl_Fig_S1_dez191
