# Supplementary material for: Dysregulation of FGFR signalling by a selective inhibitor reduces germ cell survival in human fetal gonads of both sexes and alters the somatic niche in fetal testes
Source: Hum Reprod. 2019 Nov 17;34(11):2228–43. doi: 10.1093/humrep/dez191 (PMC6994936; doi:10.1093/humrep/dez191)
Supplement: Suppl_Fig_S1_dez191 [file suppl_fig_s1_dez191.pdf]

Human Reproduction, pp. 1–1, 2019
doi:10.1093/humrep/dez191

human reproduction

SUPPLEMENTARY DATA

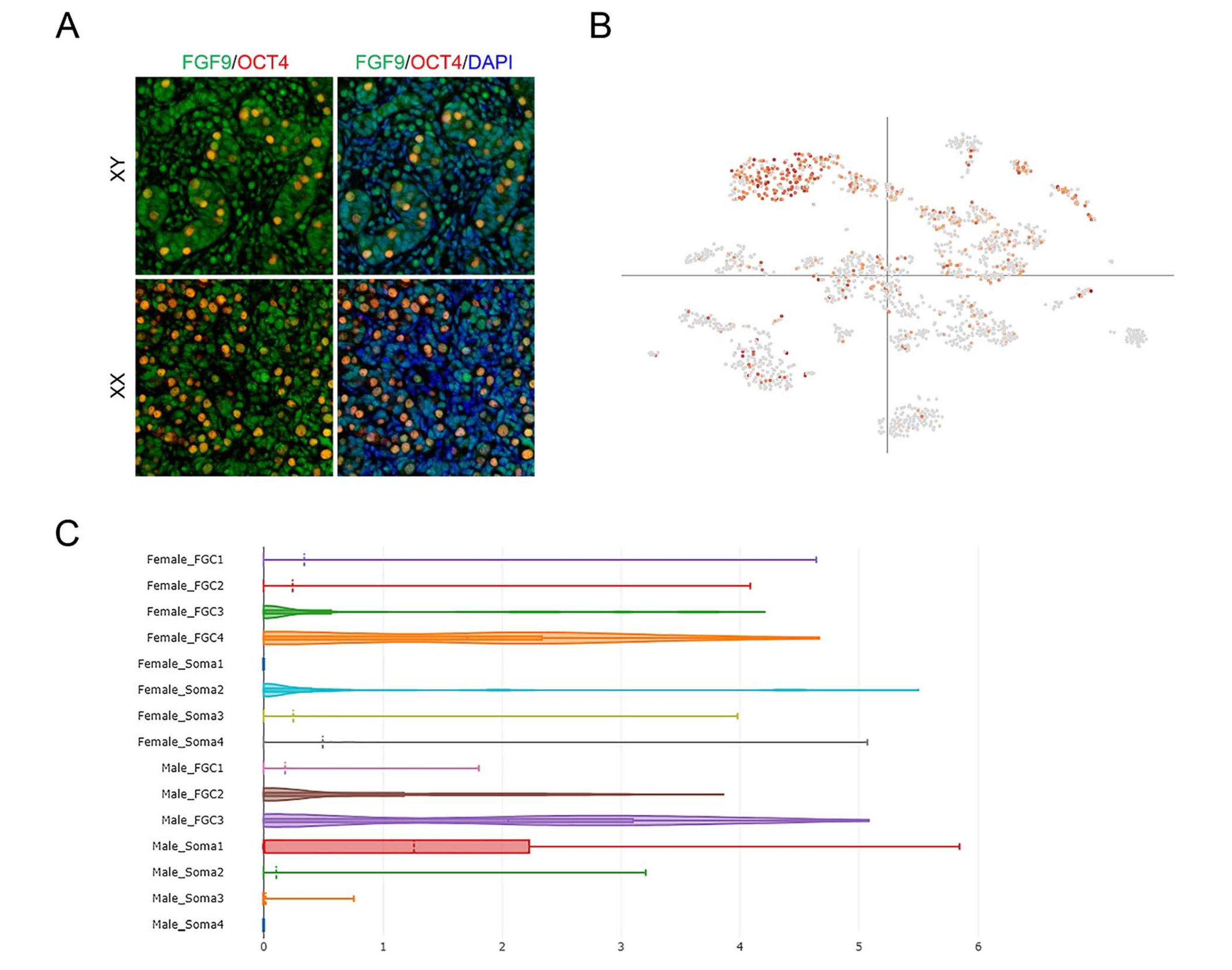

Supplementary Figure S1 Expression of FGF9 in human fetal gonads. (**A**) Immunofluorescent staining of FGF9 (green), the germ cell marker OCT4 (red) and DAPI (blue) in human fetal testes (XY) and ovaries (XX). In fetal testes, FGF9 is expressed in gonocytes (co-expressed with OCT4), Sertoli and a sub-population of interstitial cells, while in fetal ovaries FGF9 is expressed in oogonia (co-expressed with OCT4) and in a sub-population of somatic cells. (**B–C**) FGF9 expression in human fetal gonads based on scRNAseq data from Li et al. (2017). Data are illustrated using the Repro Genomics Viewer (https://rgv.genouest.org/) (Darde et al., 2015, Darde et al., 2019) to show a scatter plot and violin plot, respectively. *FGF9* is expressed in germ cells and somatic cells in human fetal testes and ovaries.
